# PIKfyve mediates the maturation of mycobacteria-containing vesicles

**DOI:** 10.3389/fcimb.2026.1792069

**Published:** 2026-04-15

**Authors:** Salomé Muñoz-Sánchez, Michiel van der Vaart, Annemarie H. Meijer

**Affiliations:** Institute of Biology Leiden, Leiden University, Leiden, Netherlands

**Keywords:** autophagy, innate immunity, mycobacterial infection, phagosome maturation, PIKfyve, zebrafish

## Abstract

Dynamic interactions between autophagosomes and lysosomes are key to ensuring the trafficking of mycobacteria through the degradative autophagolysosomal pathway. Phosphoinositides (PtdIns), play critical roles in directing the trafficking of pathogens through phagosomal or autophagosomal degradation pathways. Phosphoinositide kinase, FYVE-type zinc finger containing, PIKfyve catalyzes the phosphorylation of PtdIns(3)P to PtdIns(3,5)P_2_. Generation of PtdIns(3,5)P_2_ is critical for the maturation of autophagosomes and the proper functioning of lysosomes. The involvement of PIKfyve in immune defense against mycobacterial infections has not been addressed. Here, we studied the role of PIKfyve in *Mycobacterium marinum* (*Mm*) infection of zebrafish larvae using two chemical inhibitors of the enzymatic activity of PIKfyve, YM201636 and Apilimod. We demonstrate that PIKfyve is required for *Mm*-containing vesicle trafficking towards degradative lysosomes. We found that in the infected macrophages, PIKfyve facilitates autophagy pathway activation and (auto)phagosome maturation, and protects against cell death, thus boosting the host immune response against mycobacterial infection. This study links PIKfyve to the autolysosomal defense against a mycobacterial pathogen.

## 1 Introduction

Bacterial species belonging to the genus *Mycobacterium* comprise a number of intracellular pathogens that are notorious for causing life-threatening infectious diseases. The *Mycobacterium tuberculosis* complex (MTBC) is a genetically related group that causes tuberculosis in a wide range of hosts and includes *Mycobacterium tuberculosis* (*Mtb*), the causative agent of tuberculosis in humans (Zhang et al., 2022). In addition, *Mycobacterium marinum* (*Mm*), although not part of the MTBC, can cause tuberculosis-like disease in cold-blooded animals with similar pathology including the formation of tuberculous granulomas (Simeone et al., 2015; Ramakrishnan, 2020).

During the initial phase of infection, bacteria are taken up by macrophages through phagocytosis. Pathogenic mycobacteria subsequently create an intravesicular replication niche by inhibiting the maturation of phagosomes and the fusion with lysosomes, thus preventing exposure to the antibacterial functions of these digestive organelles (Deretic et al., 2006). In addition, these pathogens can rupture the phagosomal membrane and access the cytosol, where they become targets of the host-defensive autophagy pathway (Simeone et al., 2021). Autophagy captures cytosol-invading pathogens in a double membrane vesicle to enter them into the lysosomal degradation pathway (Deretic et al., 2013). A large body of evidence supports that autophagy restricts mycobacterial replication, and plays an important role in the host defense against tuberculosis and tuberculosis-like infections (Zhang et al., 2019; Warner et al., 2025; Lei et al., 2026). Nevertheless, mycobacteria-containing autophagosomes are not necessarily destined for degradation, as mycobacteria have evolved mechanisms to inhibit autophagy or even escape from autophagic vesicles (Bernard et al., 2020; Aylan et al., 2023). For example, *Mtb* mannose-capped lipoarabinomannan (ManLAM) present in the bacterial envelope affects autophagy initiation reducing the trafficking of the autophagy marker LC3 to mycobacteria-containing vesicles (Shui et al., 2011). Furthermore, *Mtb* induces the expression of the host microRNA miR-33,which in turn downregulates the expression of autophagy genes including ATG5, ATG12, LC3, and lysosome-associated membrane glycoprotein 1 (LAMP1), reducing autophagic flux (Ouimet et al., 2016).

The complex interactions of mycobacteria with autophagy and other cellular degradation pathways are still far from understood. Membrane phospholipids, particularly phosphoinositides (PtdIns), play critical roles in directing the trafficking of pathogens through phagosomal or autophagosomal degradation pathways (Posor et al., 2022). Through phosphorylation at the 3’, 4’, and/or 5’- hydroxyl groups of the inositol ring of phosphatidylinositol, seven different types of PtdIns are produced. These PtdIns present a local subcellular distribution on different vesicle types, and their production is highly dynamic (Vines et al., 2023). PtdIns phosphorylation by the class III phosphoinositide (PI) 3-kinase, Vps34, generates PtdIns3P, which is an extensively studied autophagy regulator, promoting the nucleation, elongation, and closure of nascent autophagosome membranes (Lystad and Simonsen, 2016; Rodgers et al., 2022). These processes are activated due to the recruitment of proteins with PtdIns3P-binding domains, such as the lipid kinase called phosphoinositide kinase, FYVE-type zinc finger containing, also known as PIKfyve or Fab1 in yeast (Krishna et al., 2016; Reggiori and Ungermann, 2017; Fountain et al., 2021).

PIKfyve has recently gained attention as a potential target for inhibiting the entry of SARS-CoV-2 and other respiratory viruses into host cells (Chatterjee and Thakur, 2022). In addition, PIKfyve has been studied in the context of several bacterial infections. *Salmonella enterica* serovar Typhimurium invades non-phagocytic cells by macropinocytosis, and PIKfyve was shown to be required for the fusion of macropinosomes with organelles of the late endosomal/lysosomal system (Kerr et al., 2010). Consequently, inhibiting PIKfyve activity interferes with the formation of the *Salmonella*-containing vacuole and limits intracellular replication of the pathogen (Kerr et al., 2010). Host cell proteoglycans play an important role in the PIKfyve-dependent endo-lysosomal fusions that sustain the intracellular niche of *Salmonella* (Galeev et al., 2020). *Coxiella burnetii* expresses several virulence factors that perturb the activity of PIKfyve, which enables the pathogen to effectively expand the *Coxiella-*containing vacuole (Martinez et al., 2016; Zhao et al., 2024). Furthermore, PIKfyve has been shown to restrict the intracellular growth of *Legionella pneumophila* by promoting the delivery of vacuolar V-ATPase and hydrolytic enzymes to the phagosome (Buckley et al., 2019). However, the role of PIKfyve in mycobacterial infections has not been addressed until now.

Here, we studied the hypothesis that PIKfyve is required for the maturation of mycobacteria-containing vesicles. To address this hypothesis in an *in vivo* context, we used *Mm* infection of zebrafish larvae, a widely used model to study mechanisms underlying the pathogenesis of tuberculosis (Cronan and Tobin, 2014; Muñoz-Sánchez et al., 2020; Varela and Meijer, 2022; Menon et al., 2025). Injecting *Mm* into the thin tissue of the tail fin allowed us to image *Mm* interaction with vesicular pathways at high resolution (Hosseini et al., 2014; Muñoz-Sánchez et al., 2023). We found that chemical inhibition of PIKfyve resulted in reduced association of *Mm* with the autophagy marker LC3 and in reduced acidification of *Mm*-containing vesicles. Furthermore, PIKfyve inhibition led to increased levels of cell death of infected macrophages, indicating that PIKfyve function is required to contain *Mm* infection intracellularly. These results support that PIKfyve mediates maturation of (auto)phagosomal *Mm*-containing vesicles, increasing the acidification, and ultimately enhancing the cell autonomous resistance to mycobacterial infection.

## 2 Materials and methods

### 2.1 Zebrafish husbandry and lines

Zebrafish (*Danio rerio*, strain AB/TL) were maintained and handled in accordance with the directives of the local animal welfare committee of Leiden University (License number 10612) and the standard guidelines from the Zebrafish Model Organism Database (https://zfin.org). Adult fish were crossed in a single couple, allowing natural fertilization at the start of the light/dark cycle (14 h light/10 h dark). Eggs were kept in egg water (60μg/mL Instant Ocean Sea salt) at 28 °C until reaching 72 hours post fertilization. The following transgenic lines were used: *Tg* (*CMV: GFP-LC3*) ( (He et al., 2009), RRID: ZFIN_ZDB-GENO-091029-2); *Tg* (*mpeg1.1:mCherry-F*) ( (Bernut et al., 2014), RRID: ZFIN_ZDB-GENO-130722-1); and *Tg* (*mpeg1:EGFP*) ( (Ellett et al., 2011), RRID: ZFIN_ZDB-GENO-120402-4), *Tg (BAC(tp63:Gal4FF);4xUAS: EGFP-FYVE)* (Rasmussen et al., 2015), (RRID: ZFIN_ZDB-GENO-150424-7;ZFIN_ZDB-GENO-150424-8), shortly referred to as EGFP-FYVE.

### 2.2 PIKfyve sequence analysis

Protein sequences were obtained from the UniProt database (Bateman et al., 2023). *Homo sapiens* (Q9Y2I7-1, UniProt) and *Danio rerio* (B2KTE4, UniProt) PIKfyve sequences ortholog alignments were done with Clustal Omega ( (Sievers et al., 2011; Sievers and Higgins, 2018), RRID: SCR_001591).

### 2.3 Drug treatment

To study the role of the lipid kinase PIKfyve during the host immune response against *Mm* infection, zebrafish larvae were treated with chemical inhibitors of PIKfyve enzymatic activity. Two structurally distinct PIKfyve inhibitors (YM201636 and apilimod) were used to pharmacologically inhibit PIKfyve activity and block the production of PtdIns(3,5)P_2_ (Jefferies et al., 2008; Cai et al., 2013). Prior to infection, zebrafish larvae were incubated for 2 hours at 28 °C in YM201636 10µM (SelleckChem, S1219) or Apilimod 1, 2, and 5 µM (Cayman Chemical, 19094). Drug treatment was sustained during the course of the infection. Dimethyl sulfoxide solution (DMSO) was used as a vehicle control.

### 2.4 *Mycobacterium marinum* infection

*Mm* M strain fluorescently labeled with E2-Crimson ( (Takaki et al., 2013), RRID: Addgene_30178) was used for infection experiments. Bacteria were cultured grown in 7H10 agar and subsequently cultured in Middlebrook 7H9 medium supplemented with ADC, 0,05% Tween-80, glycerol, and hygromicin at 28 °C. Bacteria cultures were harvested during logarithmic growth phase, washed in phosphate-buffered saline (PBS), and resuspended in PBS containing 2% Polyvinylpyrrolidone (PVP40) as previously described (Benard et al., 2012). Zebrafish larvae staged at 72 hours post fertilization (hpf) were anesthetized with tricaine and infected by tail-fin microinjection, following the protocol described previously (Muñoz-Sánchez et al., 2023). An injection of 0.5 nL containing ~100 colony forming units (CFU) was delivered per larva. Successful injection was verified by the transient formation of a localized blister-like thickening at the injection site and by fluorescence microscopy.

### 2.5 Acidic compartments labeling

Larvae were incubated for 1 hour at 28 °C in egg water with 10 µM LysoTracker Red DND-99 staining (ThermoFisher, L7528). Samples were washed in egg water and euthanized with tricaine overdose (Tricaine/MS222, Sigma-Aldrich, Darmstadt, Germany) and fixed in Pierce 4% formaldehyde (ThermoFisher, 28908) in Phosphate Buffered Saline solution (PBS) for 45 min at room temperature (RT). Finally, samples were washed in PBS and mounted for imaging.

### 2.6 *In situ* cell death detection

Infected samples were euthanized with tricaine overdose (Tricaine/MS222, Sigma-Aldrich, Darmstadt, Germany) and fixed in 4% PFA for 45 min at RT and washed in PBS. Samples passed through a dehydration/rehydration cycle in a methanol series (25%, 50%, 75%, and 100% methanol in PBS-Tween 0.1% v/v). We then permeabilized them with proteinase K 10 µg/mL (Merk, 3115836001) for 45 min at 37 °C and post-fixed in 4% PFA for 20 min at RT. Samples were then washed and incubated in *In Situ* Cell Death Detection Kit, TMR red (Roche, 12156792910) overnight at 37 °C and covered from the light. Finally, samples were washed in PBS-Tween 0.1% v/v and mounted for imaging.

### 2.7 Confocal laser scanning microscopy

Larva samples were mounted in a custom-made 2% agarose mold and covered with 1% low-melting-point agarose. After solidifying, the mold was covered with PBS and imaged with a TCS SP8 HyD confocal microscope (Leica) using a 40x oil immersion objective (NA 1.3).

### 2.8 Image analysis

Here, we employed an image-based approach to infection biology in the zebrafish model. Using the TFI technique in combination with confocal microscopy, we obtained optical access to the entire infected tissue volume, enabling three-dimensional (3D) spatial analysis of infected cells and subsequent quantitative comparisons. To extract information from these datasets, we used the bioimage analysis platform ImageJ (Schindelin et al., 2012). We quantified fluorescent signals through multiple depths along the z-axis (z-stacks), focusing on mycobacterial clusters.We defined the following 3D image analysis workflow. i) raw images were pre-processed using edge and symmetry filters implemented in the 3D ImageJ Suite plug-in (Ollion et al., 2013); ii) images were segmented using the 3D iterative thresholding algorithm (Gul-Mohammed et al., 2014); iii) segmented objects corresponding to *Mm* clusters, LC3-positive compartments, and LysoTracker-labeled lysosomes, were identified for quantitative analysis; iv) spatial relationships between these objects were analyzed using the DiAna ImageJ plugin (Gilles et al., 2017), enabling object-based colocalization and distance measurements in 3D. Output parameters included the total volume and number of segmented objects (i.e., *Mm* clusters, LC3-positive compartments, and LysoTracker-labeled lysosomes), the volume of association between *Mm* clusters and LC3-positive compartments, the colocalization volume between *Mm* clusters and LysoTracker-labeled compartments, and the minimum distance between *Mm* clusters and LC3- or LysoTracker-positive structures. All parameters were compared across experimental conditions. Surface-based three-dimensional reconstructions were generated using Blender software (https://www.blender.org).

### 2.9 Statistical analysis

All experiments were conducted at least three times independently. Statistical analysis was performed using GraphPad Prism 10 software (GraphPad, https://www.graphpad.com). Comparisons between two groups were performed using the Mann-Whitney test. For experiments with more than two groups and one independent variable, the Kruskal-Wallis test and Dunn’s multiple comparisons test were used. Experiments with two independent variables were analyzed using Two-Way ANOVA and Tukey’s multiple comparisons test. Differences between groups were considered statistically significant for p < 0.05.

## 3 Results

### 3.1 *In vivo* analysis of PIKfyve function using the zebrafish model

**Figure 1:**
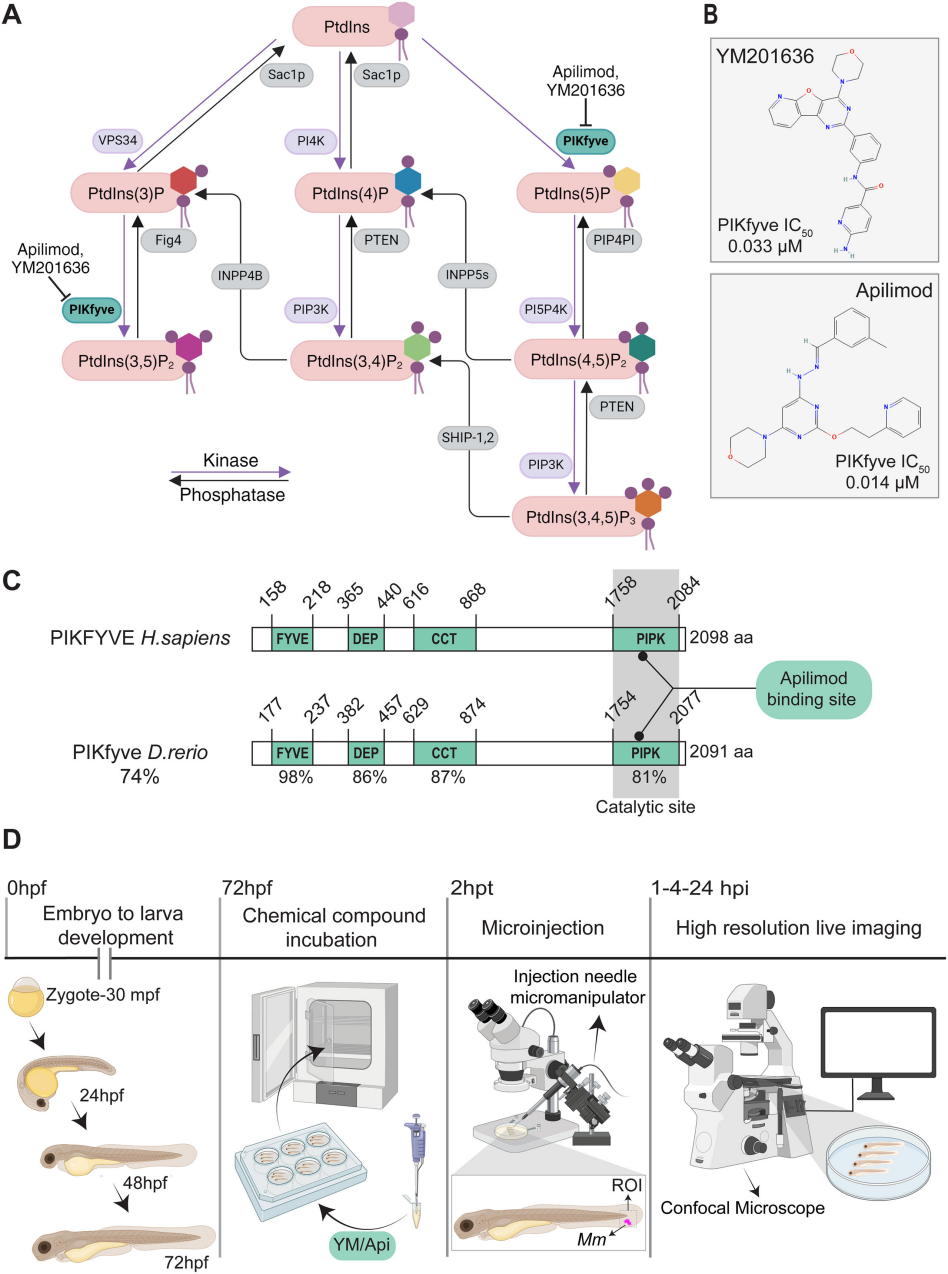
PIKfyve protein domains. **(A)** Phosphoinositide metabolism. Kinases (purple arrow) catalyse the addition of phosphate groups to the inositol ring of phosphatidylinositol and phosphatases (black arrow) catalyse their removal. PIKfyve, highlighted in green, phosphorylates PtdIns to PtdIns(5)P and PtdIns3P to PtdIns(3,5)_2_. **(B)** Chemical structures of the PIKfyve inhibitors YM201636 and Apilimod and their IC_50_ **(C)** PIKfyve protein domains. Schematic showing *H. sapiens* and *D. rerio* PIKfyve protein domains. The sequence identity of the full-length protein, binding domain, and catalytic domain is shown. Apilimod binding site is indicated. FYVE: Fab1, YOTB, Vac1, and EEA1; DEP: Dishevelled, Egl10 and Pleckstrin; CCT: Chaperonin Containing TCP1; PIPK: Phosphatidyl Inositol Phosphate Kinase. **(D)** Experimental design diagram. Zebrafish 72 hpf larvae were incubated in PIKfyve chemical inhibitor bath solution for 2 hours, after which fluorescently labeled *Mm* was microinjected into the tail fin. Live and fixed samples were mounted and imaged. CLSM was performed at the ROI at 1, 4, and 24 hpi. CLSM, Confocal laser scanning microscopy; hpf, hours post fertilization; hpi, hours post infection; hpt, hours post-treatment; ROI, region of interest.

In this study, we set out to analyze the role of PIKfyve in mycobacteria-containing vesicle maturation using the zebrafish model. PIKfyve catalyzes the phosphorylation of PtdIns to generate PtdIns(5)P and the conversion of PtdIns(3)P to PtdIns(3,5)P_2_ (**Figure 1A**). PIKfyve catalytic activity can be pharmacologically inhibited using the small molecules YM201636 (Hayakawa et al., 2006; Jefferies et al., 2008) and Apilimod (Cai et al., 2013; Gayle et al., 2017) (**Figure 1B**). We applied, these two structurally distinct inhibitors to pharmacologically inhibit PIKfyve activity, while reducing the likelihood that the observed effects are due to off-target activity of a single compound.

Considering that YM201636 and Apilimod were initially developed to inhibit human PIKfyve, we analyzed the amino acid sequence identity between the *H. sapiens* and *D. rerio* PIKfyve proteins. Full-length protein sequence alignment showed that zebrafish PIKfyve shares 74% identity with human PIKFYVE (**Figure 1C**). Protein domain sequence alignment resulted in high similarity in all the functional domains. The N-terminal five-finger binding domain (98% identity), the C-terminal PIPK catalytic domain (81% identity), the DEP intracellular protein targeting domain (86% identity), and the CCT chaperone-like domain (87% identity). Furthermore, the Apilimod binding site is conserved. The level of sequence identity suggests a functional conservation between human and zebrafish enzymes. In agreement, previous studies using YM201636 and Apilimod in zebrafish resulted in a typical cellular vacuolation phenotype, similar to that observed in human cells (Mei et al., 2022; Attia et al., 2026). Furthermore, these studies demonstrated extensive similarity between the phenotypic effects of PIKfyve genetic and chemical inhibition. To further validate the use of these chemical inhibitors, we tested the effect of the inhibitors on a zebrafish line expressing a EGFP-FYVE probe, which binds to PtdIns(3)P on early endosomes (Rasmussen et al., 2015; van der Vaart et al., 2020). PIKfyve chemical inhibition by either of the two inhibitors markedly increased the size of the EGFP-FYVE-labeled vesicles, consistent with blocked maturation of these vesicles due to PIKfyve inhibition (**Supplementary Figures 1A, B**).

**Figure 2:**
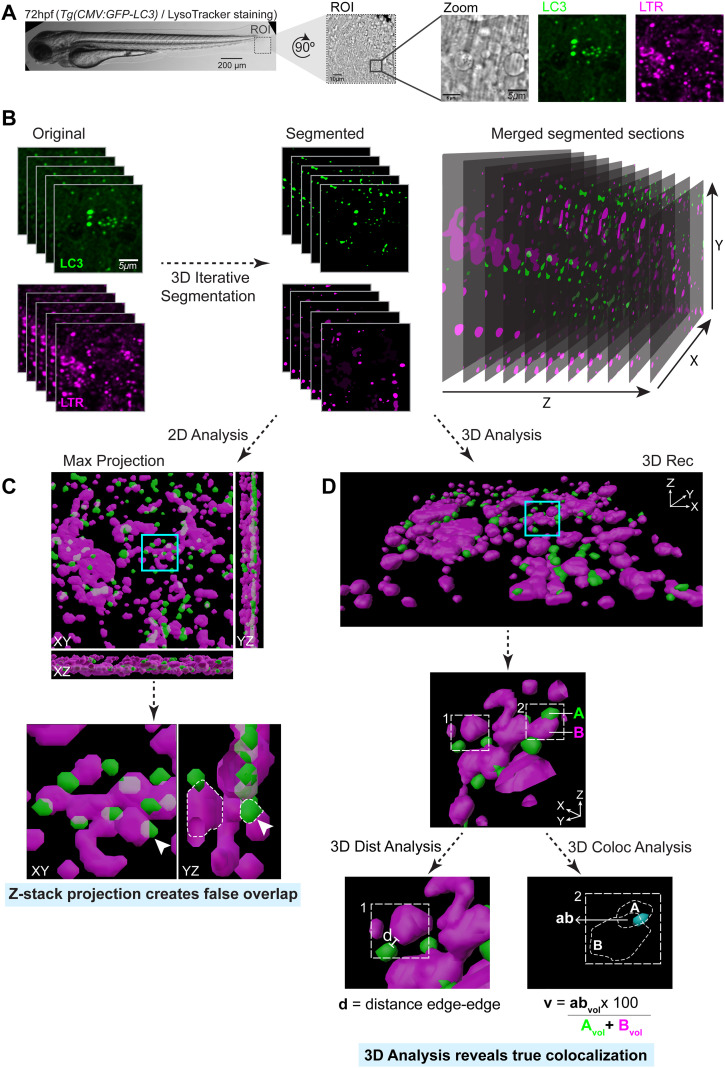
Image analysis workflow. **(A)** Representative bright-field image of a 72 hpf larva ubiquitously expressing the *CMV: GFP-LC3* construct and stained with LysoTracker. CLSM imaging was performed at the ROI in the tail fin. **(B)** Close-up of pseudo-colored image stacks from the original image and the corresponding objects after 3D iterative segmentation (green: LC3; magenta: LTR). **(C)** Two-dimensional (2D) analysis, using maximum-intensity projection of the z-stack as the labeled image, can produce false overlapping areas due to structures located at different depths. **(D)** Three-dimensional (3D) analysis, using the volume reconstruction as the labeled image, allows accurate quantification of spatial relationships between objects, retrieving the parameters d and v, where d is the distance between object edges and v is the percentage of colocalizing volume normalized to the volume of both objects. Hpf, hours post-fertilization; LTR, LysoTracker staining; ROI, region of interest.

To evaluate PIKfyve function during *Mm* infection in zebrafish, we used an experimental setup where PIKfyve chemical inhibitor treatment was applied by bath exposure at 72 hpf, 2 hours before the injection of *Mm* bacteria in the tail fin, which allows high-resolution 3D live imaging (**Figure 1D**). To analyze the acquired 3D imaging data and test the hypothesis that PIKfyve is required for the maturation of *Mm*-containing compartments, we developed a 3D image analysis workflow (**Figure 2**). The workflow was implemented using the 3D ImageJ Suite (Ollion et al., 2013) and the DiAna ImageJ plugins (Gilles et al., 2017). Confocal image stacks were segmented to identify *Mm* clusters, Lc3-positive compartments, and LysoTracker-labeled lysosomal structures, which were then used as objects for quantitative analysis. This approach enabled measurement of the spatial relationships between these structures, including the minimum distance between object surfaces (d_min_) and the fraction of overlapping volume (v). These parameters were used to determine the effect of PIKfyve inhibition on the association of the autophagy marker Lc3 and the acidification indicator LysoTracker (LTR) with *Mm*. The same 3D analysis pipeline was also applied to quantify the total volume of TUNEL signal as a measure of *Mm*-induced host cell death.

### 3.2 Effects of PIKfyve inhibition on Lc3 and LysoTracker accumulation

**Figure 3:**
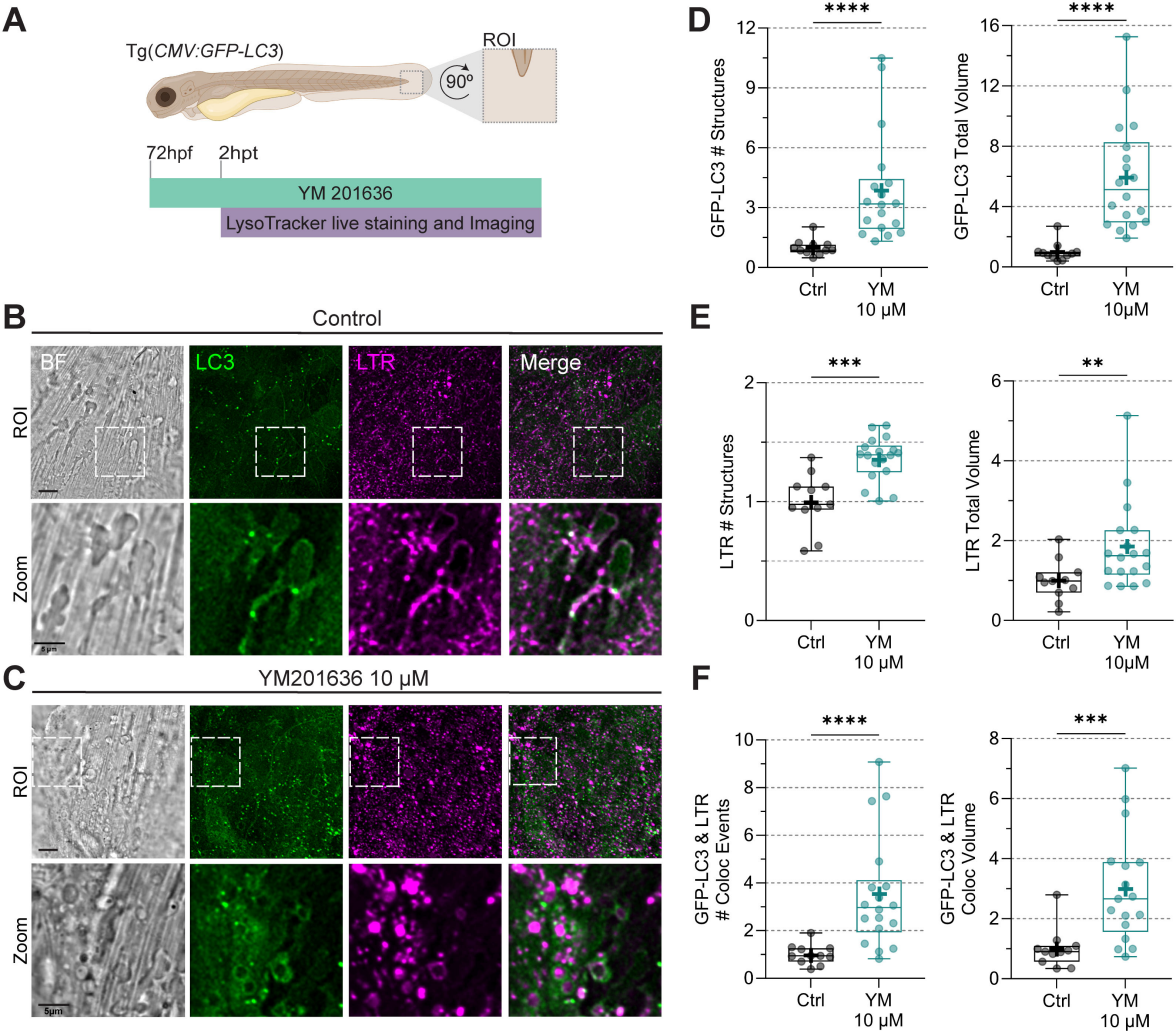
PIKfyve chemical inhibition with YM201636 causes vacuolation and increases the number of LC3-positive and acidic structures in zebrafish. **(A)** Transgenic *(CMV: GFP-LC3)* 72 hpf zebrafish larvae, labeling LC3 protein (green), were incubated in YM201636 10µM for two hours, after which LTR staining (magenta) was added. CLSM live imaging was performed at the ROI in the tail fin. **(B, C)** Representative bright field and fluorescent images. Scale bar ROI 10 µm and zoom 5 µm. **(D)** Quantification of GFP-LC3 total number of structures and volume. **(E)** Quantification of LTR total number of structures and volume. Data points correspond to the sum of structures and volumes per FOV as a fraction of the control. **(F)** Number of colocalization events and colocalization volume between LC3 and LTR. Data points correspond to the sum of colocalization events and percentages of colocalization volume per FOV as a fraction of the control. All the results were graphed in box plots from min to max, with the mean displayed as ‘+’. Statistical significance was measured by Mann-Whitney test. N = 3 independent experiments and n = 7 individuals per experiment. *: p ≤ 0.05, **: p ≤ 0.01, ***: p ≤ 0.001, ****: p ≤ 0.0001. FOV, Field of View; Hpf, hour post-fertilization; hpt, hour post-treatment; LTR, LysoTracker staining; ROI, region of interest.

To study PIKfyve function, we first used YM201636, which has been demonstrated to inhibit PIKfyve in several studies (Jefferies et al., 2008; Kim et al., 2014; Bissig et al., 2017). We used a transgenic zebrafish line ubiquitously expressing GFP-LC3 in combination with LTR live staining (**Figure 3A**). We tested YM201636 at a dose of 10 µM, based on previous work in zebrafish (van der Vaart et al., 2020). Imaging of a region of the tail fin showed extensive vacuolation in response to the drug treatment (**Figures 3B, C**), consistent with previous studies in cultured cells (Martin et al., 2013; Thieleke‐Matos et al., 2014; Wang et al., 2017). 3D analysis revealed that LC3 and LTR signals increased in the total number of structures and total volume (**Figures 3B–E**). Furthermore, the number of colocalization events between LC3 and LTR was increased, as well as their total colocalization volume (**Figure 3F**).

**Figure 4:**
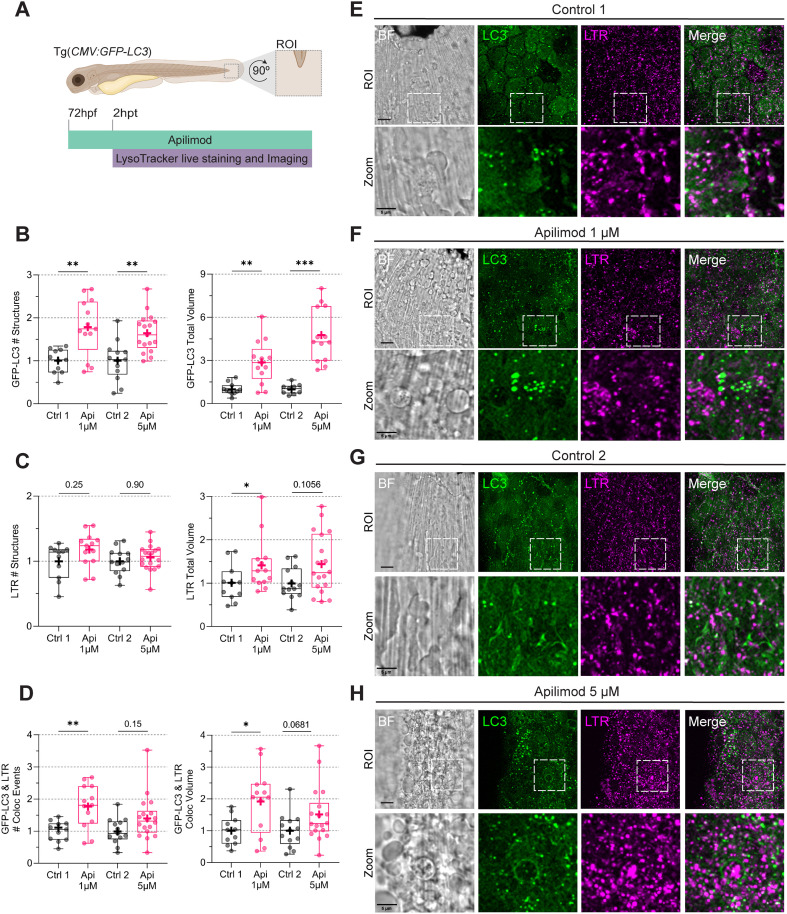
PIKfyve chemical inhibition with Apilimod causes vacuolation and increases the volume of LC3-positive and acidic structures in zebrafish. **(A)** Transgenic *(CMV: GFP-LC3)* 72 hpf zebrafish larvae, labeling LC3 protein (green), were incubated in Apilimod 1 or 5 µM for two hours, after which LTR staining (magenta) was added. CLSM live imaging was performed at the ROI in the tail fin. **(B)** Quantification of GFP-LC3 total number of structures and volume. **(C)** Quantification of LTR total number of structures and volume. Data points correspond to the sum of structures and volumes per FOV as a fraction of the control. **(D)** Number of colocalization events and colocalization volume between LC3 and LTR. Data points correspond to the sum of colocalization events and percentages of colocalization volume per FOV as a fraction of the control. All the results were graphed in box plots from min to max, with the mean displayed as ‘**+**’. Statistical significance was measured by Mann-Whitney. N = 3 and n = 7 *: p ≤ 0.05, **: p ≤ 0.01, ***: p ≤ 0.001, ****: p ≤ 0.0001. **(E–H)** Representative bright field and fluorescent images. Scale bar ROI 10 µm and zoom 5 µm. FOV, Field of View; Hpf, hour post-fertilization; hpt, hour post-treatment; LTR, LysoTracker staining; ROI, region of interest.

To confirm these observations, we used Apilimod (Jefferies et al., 2008; Cai et al., 2013), which has been reported as a more specific and selective PIKfyve inhibitor (**Figure 4A**). We tested Apilimod at two doses, 1 and 5 µM. Like YM201636, Apilimod induced vacuolation, which was more extensive at the 5 µM dose, disturbing the tissue integrity (**Figures 4E–H**). Both concentrations, 1 and 5 µM, increased the number of LC3 structures and their total volume (**Figures 4B, E-H**), in line with the result of YM201636 treatment. However, for LTR, we only observed an increase in total volume at 1 µM (**Figure 4C**). In addition, the number of colocalization events between LC3 and LTR and their total colocalization volume were increased at 1 µM (**Figure 4D**). We assumed that the tissue disturbances caused by the Apilimod 5 µM dose may explain its reduced effects on LTR accumulation and LC3-LTR colocalization. We therefore used the lower treatment concentration of 1 µM for our further studies.

### 3.3 Effect of PIKfyve inhibition on Lc3 association with mycobacteria

**Figure 5:**
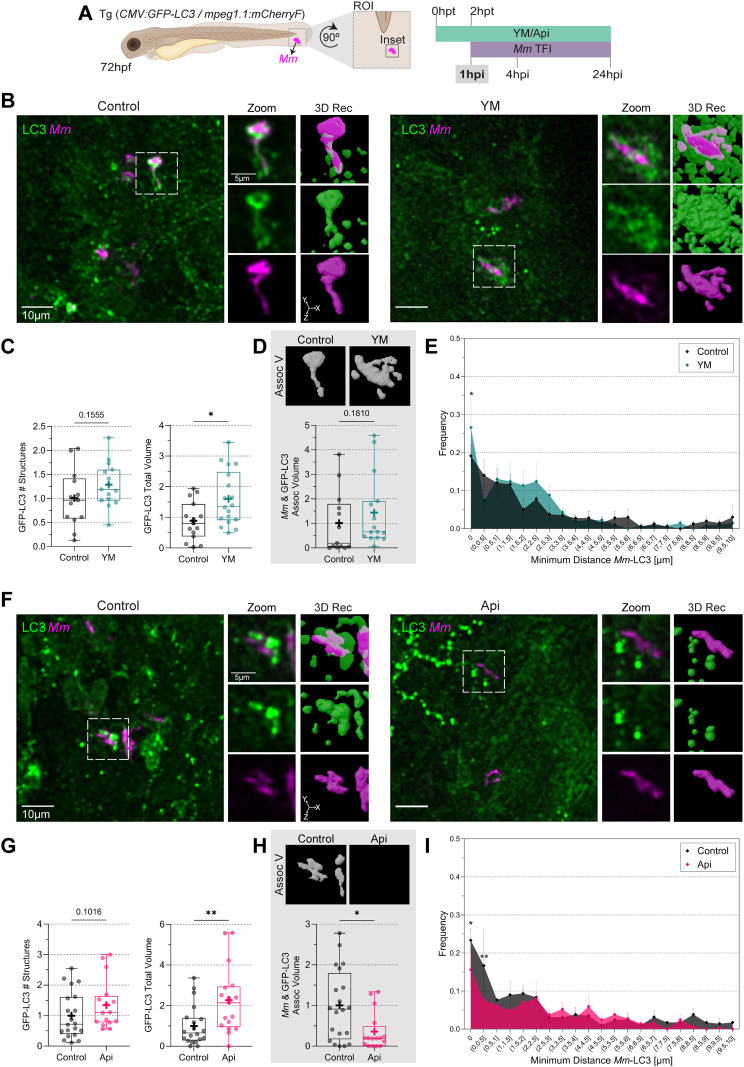
Apilimod reduces *Mm*-LC3 association volume during the initial stage of infection. **(A)** Double transgenic *(CMV: GFP-LC3/mpeg1.1:mCherryF)* 72 hpf zebrafish larvae, labeling LC3 protein (green) and macrophages (cyan), were incubated in YM201636 (YM) 10 µM or Apilimod (Api) 2 µM. After two hours, the larvae were infected with 100 CFU of E2-Crimson-labeled *Mm* (magenta). Samples were fixed at 1hpi, and CLSM imaging was performed at the ROI in the tail fin. **(B, F)** Representative fluorescent images of the inset area and 3D reconstruction of the zoomed region. Scale bar inset: 10 µm; zoom: 5 µm. **(C, G)** Quantification of GFP-LC3 total number of structures and volume. Data points correspond to the sum of structures and volumes per FOV as a fraction of the control. **(D, H)** Calculation of *Mm*-LC3 association volume. Data points correspond to the sum of percentages of association volume per FOV as a fraction of the control. The results were graphed in box plots from min to max, mean was displayed as ‘**+**’. Statistical significance was measured by Mann-Whitney test. **(E, I)** minimum *Mm*-LC3 distance frequency. Data points correspond to the counts of d_min_ as a fraction of the control. The results were graphed in frequency histograms and represented as mean ± SEM. The Two-Way ANOVA test and Tukey’s multiple comparisons test measured statistical significance. YM: N = 3 and n = 5; Api: N = 3 and n = 6. *: p ≤ 0.05, **: p ≤ 0.01, ***: p ≤ 0.001, ****: p ≤ 0.0001. d_min_: minimum distance; FOV, Field of View; Hpf, hour post-fertilization; hpt, hour post-treatment; ROI, region of interest.

**Figure 6:**
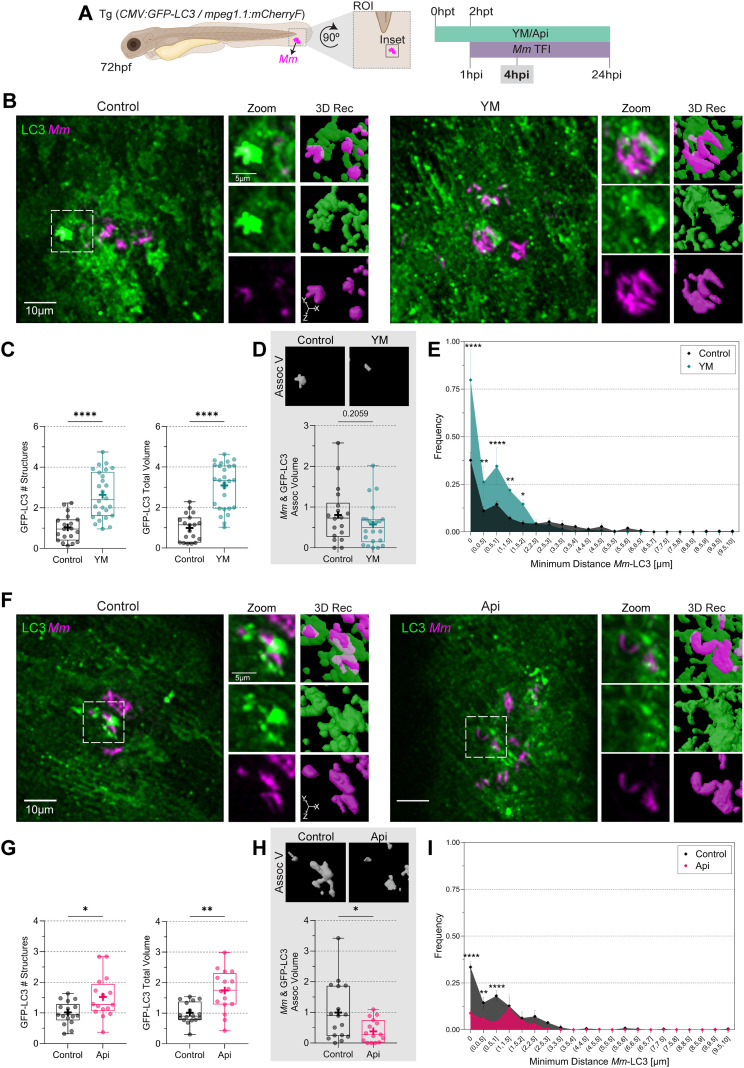
PIKfyve inhibition alters LC3 distribution in *Mm*-infected cells. **(A)** Double transgenic (CMV: GFP-LC3/mpeg1.1:mCherryF) 72 hpf zebrafish larvae, labeling LC3 protein (green) and macrophages (cyan), were incubated in YM201636 (YM) 10 µM or Apilimod (Api) 2 µM. After two hours, the larvae were infected with 100 CFU of E2-Crimson-labelled *Mm* (magenta). Samples were fixed at 4hpi, and CLSM imaging was performed at the ROI in the tail fin. **(B, F)** Representative fluorescent images of the inset area and 3D reconstruction of the zoomed region. Scale bar inset: 10 µm; zoom: 5 µm. **(C, G)** Quantification of GFP-LC3 total number of structures and volume. Data points correspond to the sum of structures and volumes per FOV as afraction of the control. **(D, H)** Calculation of *Mm*-LC3 association volume. Data points correspond to the sum of percentages of association volume per FOV as a fraction of the control. The results were graphed in box plots from min to max, mean was displayed as ‘+’. Statistical significance was measured by Mann-Whitney test. **(E, I)** minimum *Mm*-LC3 distance frequency. Data points correspond to the counts of d_min_ as a fraction of the control. The results were graphed in frequency histograms and represented as mean ± SEM. The Two-Way ANOVA test and Tukey’s multiple comparisons test measured statistical significance. YM: N = 4 and n = 5; Api: N = 4 and n = 5. *: p ≤ 0.05, **: p ≤ 0.01, ***: p ≤ 0.001, ****: p ≤ 0.0001. d_min_: minimum distance; FOV, Field of View; Hpf, hour post-fertilization; hpt, hour post-treatment; ROI, region of interest.

**Figure 7:**
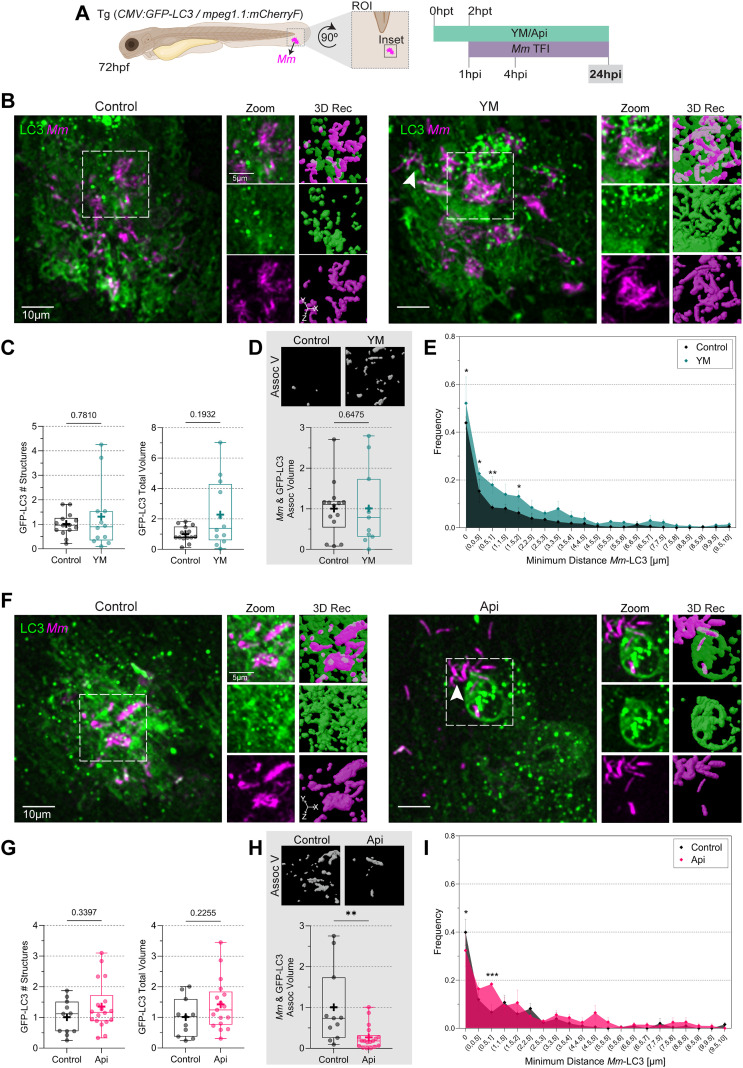
PIKfyve inhibition accelerates the appearance of extracellular *Mm* clusters. **(A)** Double transgenic (CMV: GFP-LC3/mpeg1.1:mCherryF) 72 hpf zebrafish larvae, labeling LC3 protein (green) and macrophages, were incubated in YM201636 (YM) 10 µM or Apilimod (Api) 2 µM. After two hours, the larvae were infected with 100 CFU of E2-Crimson-labeled *Mm* (magenta). Samples were fixed at 24hpi, and CLSM imaging was performed at the ROI in the tail fin. **(B, F)** Representative fluorescent images of the inset area and 3D reconstruction of the zoomed region. White arrowheads point to extracellular bacteria. Scale bar inset: 10 µm; zoom: 5 µm. **(C, G)** Quantification of GFP-LC3 total number of structures and volume. Data points correspond to the sum of structures and volumes per FOV as a fraction of the control. **(D, H)** Calculation of *Mm*-LC3 association volume. Data points correspond to the sum of percentages of association volume per FOV as a fraction of the control. The results were graphed in box plots from min to max, mean was displayed as ‘+’. Statistical significance was measured by Mann-Whitney test. **(E, I)** minimum *Mm*-LC3 distance frequency. Data points correspond to the counts of d_min_ as a fraction of the control. The results were graphed in frequency histograms and represented as mean ± SEM. The Two-Way ANOVA test and Tukey’s multiple comparisons test measured statistical significance. YM: N = 3 and n = 5; Api: N = 3 and n = 6. *: p ≤ 0.05, **: p ≤ 0.01, ***: p ≤ 0.001, ****: p ≤ 0.0001. d_min_: minimum distance; FOV, Field of View; Hpf, hour post-fertilization; hpt, hour post-treatment; ROI, region of interest.

Next, we evaluated how YM201636 and Apilimod affected the LC3-association dynamics during *Mm* infection in the tail fin. We imaged the infected area at 1hpi (**Figure 5**), 4hpi (**Figure 6**), and 24hpi (**Figure 7**). Using the 3D image analysis workflow (**Figure 2**), we first verified that at 1 hour after the initial infection, macrophage recruitment and phagocytosis of bacteria were equivalent between the control and treatment groups. At 1 hpi, no differences were found for either treatment (**Supplementary Figures 2B, C, G, H**). We then examined LC3-association with *Mm*-containing vesicles. YM201636 treatment increased the LC3 total volume but not the LC3 number of structures or the *Mm*-LC3 association volume (**Figures 5B–D**). Furthermore, we measured the minimum distance (d_min_), which is the distance between *Mm* and the nearest LC3 structure, and calculated its frequency. Treatment with YM201636 resulted in a higher frequency of LC3 structures in contact with *Mm*, d_min_ = 0 µm, compared to the control, and no differences for the structures adjacent to the *Mm* clusters, d_min_ > 0 µm (**Figure 5E**). In the same way as YM201536, Apilimod treatment also increased the LC3 total volume but reduced the *Mm*-LC3 association volume (**Figures 5F–H**). Congruent with these observations, under Apilimod treatment, we observed a lower frequency of LC3 structures in contact with *Mm*, and adjacent to the *Mm* clusters, 0 < d_min_ ≤ 0.5 µm (**Figure 5I**) compared to the control.

At 4 hpi, the effects of Apilimod and YM201636 were similar, reaching significant increases in the number of LC3 structures and LC3 total volume (**Figures 6B, C, F, G**). Similar to 1 hpi, Apilimod reduced the *Mm*-LC3 association volume (**Figure 6H**) and frequency of LC3 structures in contact and near the *Mm* clusters, 0 < d_min_ ≤ 1 µm (**Figure 6I**). Conversely, the YM201636 treatment showed higher frequencies of LC3 structures in contact and near the *Mm* clusters, 0 < d_min_ ≤ 2 µm (**Figure 6E**), with a shorter distance to the closest LC3 structure (**Supplementary Figure 3C**). This might be explained by the overall higher LC3 levels induced by this drug.

Finally, at 24 hpi, Apilimod still showed a significant reduction of the *Mm*-LC3 association volume and frequency of LC3 structures in contact with *Mm* (**Figures 7F, H, I**). This was not the case for LC3 structures and LC3 total volume, where differences were no longer observed for any treatment (**Figures 7C, G**). The YM201636 treatment had the same trend in frequency of the d_min_, but with less magnitude (**Figures 7B, E**). Although there were no differences in bacterial burden at 24hpi (**Supplementary Figures 4B, E**), we did observe differences in the presence of extracellular clusters, which were spotted only in drug-treated samples and not in control samples (**Figures 7B, F** arrowheads). In conclusion, the time series showed that Apilimod reduced LC3-association with *Mm*-containing vesicles more efficiently than YM201636 and that the effects were most prominent at 4 hpi.

### 3.4 PIKfyve inhibition reduced mycobacteria-containing vesicles maturation

**Figure 8:**
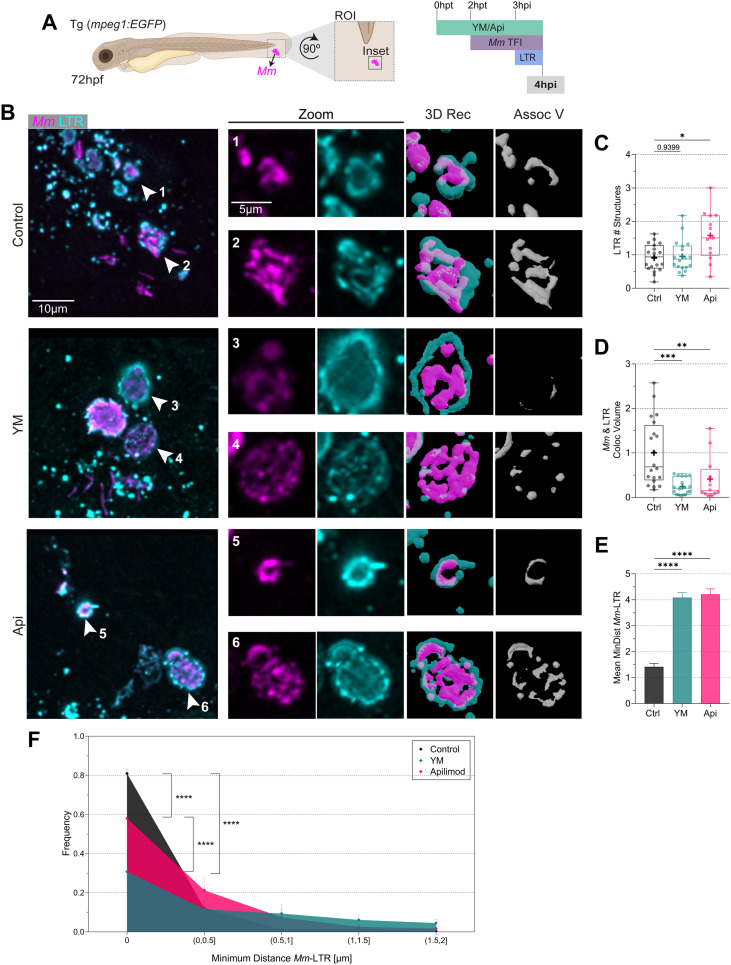
PIKfyve inhibition hinders *Mm*-containing vesicle maturation. **(A)** Transgenic *(mpeg1:EGFP)* 72 hpf zebrafish larvae were incubated in YM201636 (YM) 10 µM or Apilimod (Api) 2 µM. After two hours, the samples were infected with 100 CFU of E2-Crimson-labeled *Mm* (magenta). Samples were incubated for1 hour in LTR staining (cyan) and fixed at 4hpi. CLSM imaging was performed at the ROI in the tail fin. **(B)** Representative fluorescent images of the inset area and 3D reconstruction of the zoomed region. Scale bar inset: 10 µm; zoom: 5 µm. **(C)** Quantification of LTR total number of structures. Data points correspond to the sum of structures per FOV as a fraction of the control. **(D)** Calculation of *Mm*-LTR colocalization volume. Data points correspond to the sum of percentages of colocalization volume per FOV as a fraction of the control. The results were graphed in box plots from min to max, mean was displayed as ‘+’. Statistical significance was measured by Kruskal-Wallis and Dunn’s multiple comparisons tests. **(E)** Mean minimum *Mm*-LTR distance. Data points are measured d_min_ per FOV as a fraction of the control. The results were graphed in columns and represented as mean ± SEM. **(F)** minimum *Mm*-LTR distance frequency. Data points correspond to the counts of d_min_ as a fraction of the control. The results were graphed in frequency histograms and represented as mean ± SEM. The Two-Way ANOVA test and Tukey’s multiple comparisons test measured statistical significance. YM: N = 3 and n = 6; Api: N = 3 and n = 5. *: p ≤ 0.05, **: p ≤ 0.01, ***: p ≤ 0.001, ****: p ≤ 0.0001. d_min_: minimum distance; FOV, Field of View; Hpf, hour post-fertilization; hpt, hour post-treatment; LTR, LysoTracker staining; ROI, region of interest.

Having shown that PIKfyve inhibition reduced *Mm*-LC3 association, we proceeded to analyze its effect on *Mm*-containing vesicle acidification. Considering that the maximum effects of PIKfyve inhibition on LC3 association were observed at 4hpi, we used this time point to label acidic compartments with LTR. Apilimod but not YM201636 showed a higher number of LTR structures (**Figure 8C**), whereas for both drugs, LTR total volume was not affected (**Supplementary Figure 5B**). Colocalization analysis indicated a decrease in *Mm*-LTR colocalization volume for both treatments (**Figure 8D**). In accordance with this, we found a lower frequency of LTR structures in contact with *Mm*, d_min_ = 0 µm, and no differences for the structures near the *Mm* clusters, d_min_ > 0 µm. In conclusion, PIKfyve activity inhibition diminished *Mm*-containing vesicle acidification, which is a proxy for a reduced maturation state.

### 3.5 PIKfyve inhibition increased mycobacteria-infected cells death

**Figure 9:**
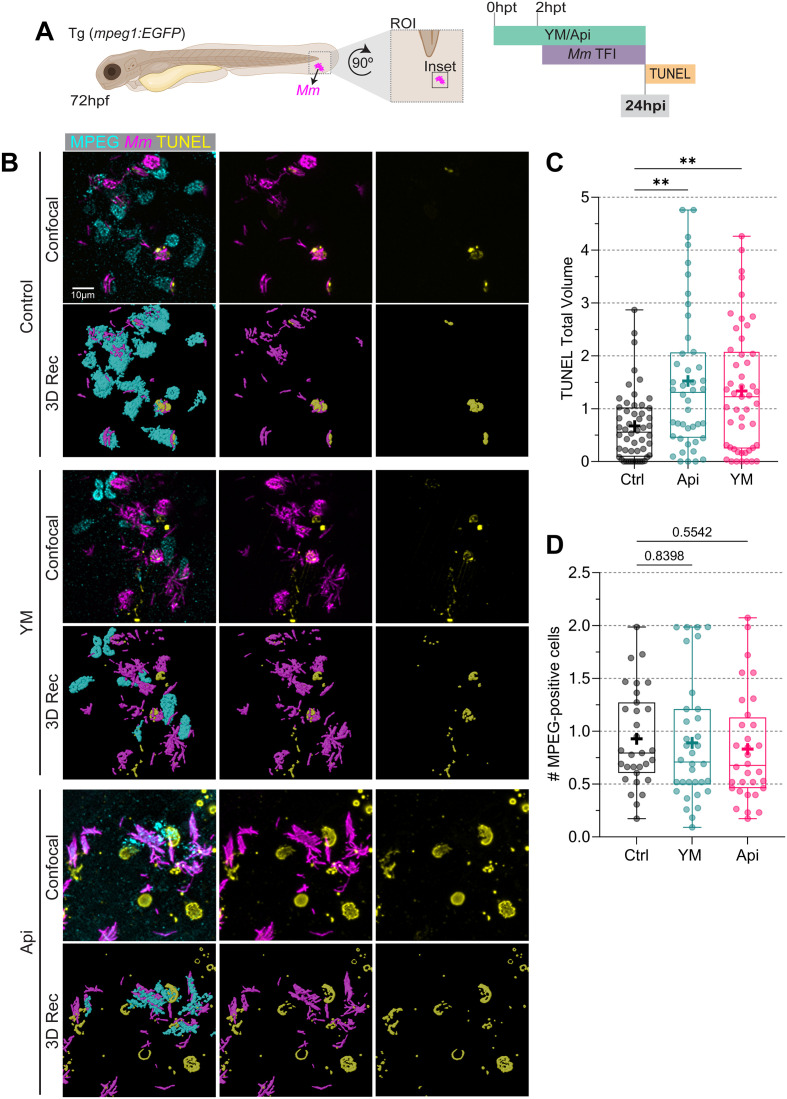
PIKfyve inhibition exacerbates *Mm*-infected cell death. **(A)** Transgenic *(mpeg1:EGFP)* 72 hpf zebrafish larvae, labeling macrophages (cyan), were incubated in YM201636 10 µM or Apilimod 2 µM. After two hours, samples were infected with 100 CFU of E2-Crimson-labeled *Mm* (magenta). Larvae were fixed at 24hpi, after which the TUNEL assay was performed (yellow). Samples were imaged by CLSM at the ROI in the tail fin. **(B)** Representative fluorescent images of the inset area and 3D reconstruction. Scale bar 10 µm. **(C)** Quantification of TUNEL total volume. Data points correspond to the sum of volumes per FOV as a fraction of the control. **(D)** Number of Mpeg-positive cells. Data points correspond to EGFP-positive cells per FOV as fraction of the control. The results were graphed in box plots from min to max, mean was displayed as ‘+’. Statistical significance was measured by Kruskal-Wallis and Dunn’s multiple comparisons tests. YM: N = 7 and n = 5; Api: N = 6 and n = 5. *: p ≤ 0.05, **: p ≤ 0.01, ***: p ≤ 0.001, ****: p ≤ 0.0001. FOV, Field of View; Hpf, hour post-fertilization; hpt, hour post-treatment; ROI, region of interest; TUNEL, TdT-mediated dUTP-X nick end labeling.

Since inhibition of PIKfyve activity reduced LC3-association and acidification of *Mm*-containing vesicles, we asked if this would also affect host cell death caused by the infection. We assessed cell death at 24 hpi, at which time point extracellular clusters of *Mm* had been observed (**Figures 7B, F**). Both YM201636 and Apilimod treatments did not affect the number of recruited macrophages nor the bacterial burden (**Figure 9D**; **Supplementary Figure 6B**). The TUNEL cell death detection assay indicated that both drugs increased cell death in the presence of bacteria (**Figures 9B, C**). This result suggests that *Mm*-infected cells succumbed to death in greater numbers in the absence of PIKfyve activity.

## 4 Discussion

Deciphering the details of host-pathogen interactions that determine the effectiveness of mycobacteria in neutralizing host immune defenses is critical for developing new therapeutic strategies (Kilinç et al., 2021). The autophagy pathway is one of these defenses, especially efficient at targeting mycobacteria when they obtain access to the cytosol of their host cell. These cytosolic invaders are engulfed in double-membrane vesicles, autophagosomes, that are delivered to lysosomes for degradation (Deretic, 2014). Dynamic interactions between autophagosomes and lysosomes are key to ensuring the trafficking of mycobacteria through the degradative autophagolysosomal pathway. Here, we demonstrate that PIKfyve is required for *Mm*-containing vesicle trafficking in the autolysosomal degradation pathway. We found that in the infected macrophages, PIKfyve facilitates autophagy pathway activation and (auto)phagosome maturation, and protects against cell death, thus boosting the host immune response against mycobacterial infection.

Proper levels of PtdIns(3,5)P_2_ are critical for the regulation of autophagy (Dall'Armi et al., 2013; Jarocki et al., 2024). Therefore, we investigated PIKfyve, the kinase that binds PtdIns(3)P via its FYVE domain and phosphorylates it, thereby exclusively controlling PtdIns(3,5)P_2_ synthesis inside the cell. We demonstrated the role of PIKfyve in *Mm* infection of zebrafish larvae using two chemical activity inhibitors, YM201636 and Apilimod. We observed enlarged vacuoles and lysosomes all around the body. This same phenomenon has been observed in zebrafish PIKfyve loss-of-function mutants, indicating that this is an actual effect of PIKfyve deficiency rather than a drug side effect (Mei et al., 2022). In addition, the same phenotype has been described previously in yeast and mice when genetically suppressing Fab1 and PIKfyve, respectively (Bonangelino et al., 2002; Duex et al., 2006; Zhang et al., 2007). The similarity of these phenotypes with lysosomal storage disorders has been previously noted (Idol et al., 2014; Berg et al., 2016; Bao et al., 2021; Xia et al., 2024), and PIKfyve function has also been implicated in vacuolation during prion diseases (Lakkaraju et al., 2021). We also observed the accumulation of LC3-positive structures, an effect that was strongest with YM201636 and could be related to the broader kinase inhibition profile of this drug (Jefferies et al., 2008; Cai et al., 2013). The accumulation of LC3-positive structures is in line with studies where PIKfyve inhibition with YM201636 resulted in autophagosome accumulation in cultured murine neurons (Martin et al., 2013) and with the results of siRNA-mediated knockdown of PIKfyve in HeLa cells (De Lartigue et al., 2009). The identity of these LC3-positive structures remains to be established, but it has been suggested they represent amphisomes, a fusion between an autophagosome and a late endosome (Mei et al., 2022). However, we frequently observed that these structures colocalized with LTR, suggesting that PIKfyve inhibition does not prevent acidification but halts vesicles in a later stage of the autolysosomal pathway.

During *Mm* infection, YM201636 and Apilimod consistently reduced *Mm*-LC3 association and *Mm*-LTR colocalization, indicating that PIKfyve is required for the autophagic targeting and acidification of *Mm*. PIKfyve is known to catalyse two steps in PtdIns formation, namely the phosphorylation of PtdIns to PtdIns(5)P and the phosphorylation of PtdIns(3)P to PtdIns(3,5)P_2_, which are both essential for autophagy regulation. The effects of PIKfyve inhibitors on *Mm*-containing vesicles could be mainly related to reduced PtdIns(3,5)P_2_ production, as PtdIns(3,5)P_2_ is known to drive autophagosome-lysosome fusions (Rusten et al., 2007; Dove et al., 2009; Ferguson et al., 2009). However, reduced production of PtdIns(5)P could also impact *Mm*-LC3 associations, as PtdIns(5)P has been shown to regulate autophagosome biogenesis (Vicinanza et al., 2015). This possibility is particularly interesting because other pathogens have been shown to increase PtdIns(5)P levels (Marcus et al., 2001; Niebuhr et al., 2002). Furthermore, it is important to note that the effects of YM201636 and Apilimod during infection could reach beyond autophagy, as PIKfyve inhibition has been shown to impair central innate immunity signaling pathways (Kawasaki et al., 2013).

*Mm* and *Mtb* are closely related pathogens that share virulence mechanisms, including the type VII secretion system ESX-1, which mediates the permeabilization of mycobacteria-containing vesicles and enables invasion of the cytosol (Menon et al., 2025). As technical limitations prevented us from assessing the possible impact if PIKfyve on vesicle permeabilization or cytosolic invasion, our study focused on the vesicle trafficking pathways. While our results support that PIKfyve has a defensive function against *Mm* infection by promoting autophagosome maturation and preventing infected cell death, it remains to be established if these results can be extrapolated to *Mtb*. To the best of our knowledge, there are currently no reports on PIKfyve inhibition during *Mtb* infection, but PIKfyve has been implicated in the host defense against two other intracellular pathogens, *C. burnetii* (Martinez et al., 2016; Zhao et al., 2024) and *L. pneumophilia* (Buckley et al., 2019). In contrast, in *S*. Typhimurium infection, PIKfyve inhibition restricted the expansion of the pathogen-containing vacuoles, indicating that this pathogen exploits PIKfyve activity to transform the phagosome into an intracellular replicative niche (Kerr et al., 2010; Galeev et al., 2020). That PIKfyve inhibition during *Mm* infection disrupts the autolysosomal degradation pathway rather than affecting the replicative niche, might be because *Mm*, similar to *Mtb*, can prevent the accumulation of the PIKfyve substrate PtdIns3P on phagosomal membranes (Fratti et al., 2003).

Altogether, the studies on different intracellular pathogens show that PIKfyve inhibition has distinct outcomes on phagosomal and autophagosomal processes (Kerr et al., 2010; Martinez et al., 2016; Buckley et al., 2019; Galeev et al., 2020; Zhao et al., 2024). However, in all cases, these outcomes could at least partly be due to impairment of lysosomal function (Choy et al., 2018; Sharma et al., 2019; Saffi et al., 2021). For example, PIKfyve inhibition has been shown to disrupt lysosomal fission and enlargement of the *C. burnetti*-vacuole, while in *Mm* infection, PIKfyve inhibition impaired acidification, presumably due to reduced fusion of lysosomes with *Mm*-containing autophagosomes or other *Mm*-containing vesicles. This notion is consistent with real-time imaging results in the zebrafish epidermis showing that PIKfyve inhibition disrupts the fusion between FIVE-labeled early endosomes and vesicles of late endosomal or lysosomal identity that were fluorescently marked with DRAM1, a crucial regulator of antimicrobial autophagy (van der Vaart et al., 2020).

The ultimate effect of PIKfyve inhibition that we observed was an increase in infected cell death, which could be a direct result of the impaired autolysosomal control of the infection. However, other PIKfyve-dependent processes could also be involved, considering that PIKfyve inhibition has been linked to various cell death processes in cancer cells as well as pluripotent stem cells (Chakraborty et al., 2022; Roy et al., 2024). The cell death of *Mm*-infected cells detected by TUNEL assay could be apoptosis or a lytic form of cell death, such as pyroptosis, an inflammation-regulated cell death mode (Kari et al., 2022). We favor the hypothesis that pyroptosis is the main cell death mechanism in this case because we observed the extracellular release of *Mm* and have previously shown that inhibition of pyroptosis reduces *Mm* bacterial burden in the zebrafish model (Zhang et al., 2020). In addition, necroptosis could be involved, which is induced under high inflammatory conditions in *Mm*-infected zebrafish (Roca et al., 2019, 2022). Similarly, *Mtb* has been shown to induce lytic forms of cell death, which enables growth of the pathogen on the cellular debris and leads to increased infection levels (Mahamed et al., 2017). On the other hand, apoptosis is viewed as a strategy of the host to eliminate the replicative niche of the pathogen. Based on our results in the current and previous work, we propose that PIKfyve is required to protect cells against host detrimental cell death triggered by *Mm* infection.

In conclusion, this study demonstrates that PIKfyve functions in the autolysosomal defense against a mycobacterial pathogen. Furthermore, we revealed a protective role for PIKfyve against *mycobacterium*-infected cell death. These results, obtained using the zebrafish larva as an *in vivo* model, add to the repertoire of PIKfyve functions that have emerged from studies in cell culture and mammalian models. Interestingly, dysregulation of PIKfyve-dependent endolysosomal pathways has also been implicated in other pathological contexts, including neurodegenerative diseases and cancer. For example, inhibition of PIKfyve has been shown to ameliorate pathology in models of amyotrophic lateral sclerosis (ALS) (Hung et al., 2023), while elevated PIKfyve expression correlates with poor prognosis and reduced response to immune checkpoint blockade therapy in cancer patients (Bao et al., 2023). In addition, pharmacological targeting of PIKfyve can enhance antitumor immune responses and improve immunotherapy efficacy in preclinical cancer models (Qiao et al., 2021). In the context of infectious disease, PIKfyve inhibitors are extensively explored as therapeutics for preventing viral entry into host cells (Nelson et al., 2017; Kang et al., 2020; Drewry et al., 2022; Baker et al., 2024; Seo et al., 2024). Based on our results, PIKfyve inhibition would clearly be non-therapeutic for mycobacterial infection. Instead, therapeutic strategies that boost PIKfyve activity would be worth investigating and could benefit from the insights on PIKfyve function obtained in this study. Future studies will be required to determine whether alterations in PIKfyve expression or activity may also have diagnostic or prognostic relevance in tuberculosis.

## Data Availability

The raw data supporting the conclusions of this article will be made available by the authors, without undue reservation.
