# Supplementary material for: PIKfyve mediates the maturation of mycobacteria-containing vesicles
Source: Front Cell Infect Microbiol. 2026 Apr 15;16:1792069. doi: 10.3389/fcimb.2026.1792069 (PMC13125058; doi:10.3389/fcimb.2026.1792069)
Supplement: Supplementary file 1 [file DataSheet1.pdf]

Supplementary Material

1 Supplementary Figures

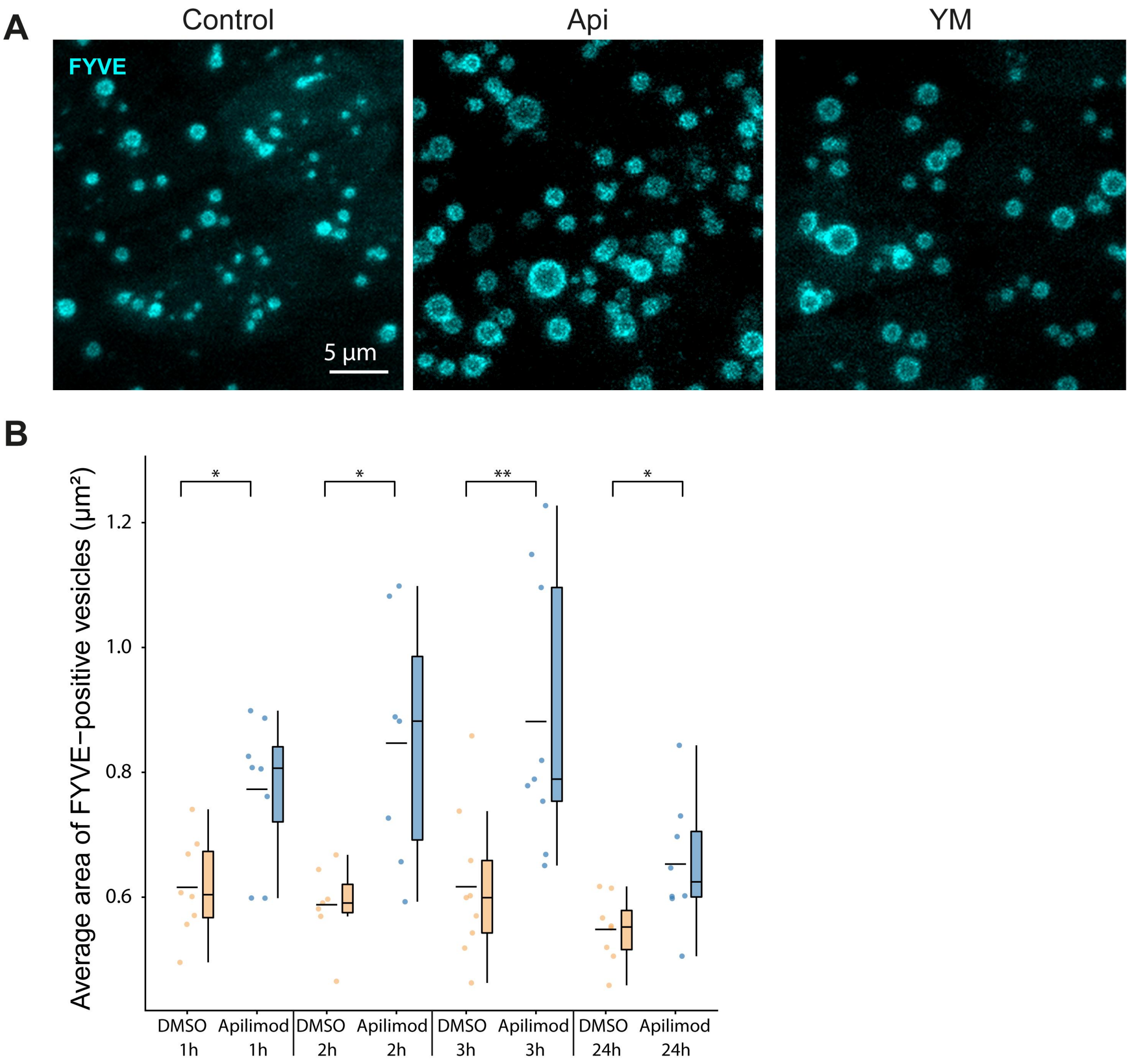

Supplementary Figure 1: Chemical inhibition of PIKfyve increases EGFP-FYVE-labeled vesicle size. *Tg(BAC(tp63:Gal4FF));4xUAS:EGFP-FYVE)* zebrafish larvae, which express a fluorescent FYVE reporter under control of a basal epithelial cell-specific promoter, were used to demonstrate the effect of PIKfyve inhibition on vesicle morphology (Rasmussen et al., 2015, van der Vaart et al., 2020). **(A)** Zebrafish larvae (3 dpf) expressing EGFP-FYVE were treated for 2 hours with 5 µm apilimod, 10 µm YM201636, or DMSO as a solvent control. Representative maximum intensity Z-projection of EGFP-FYVE in basal cell layer epithelial cells. **(B)** Zebrafish larvae (3 dpf) expressing EGFP-FYVE were treated for 1, 2, 3, or 24 hours prior to fixation and imaging with 5 µm apilimod or DMSO as a solvent control. The average area of EGFP-FYVE labeled vesicles per cell was measured using Fiji/ImageJ. $N \geq 7$ individual zebrafish larvae per group.

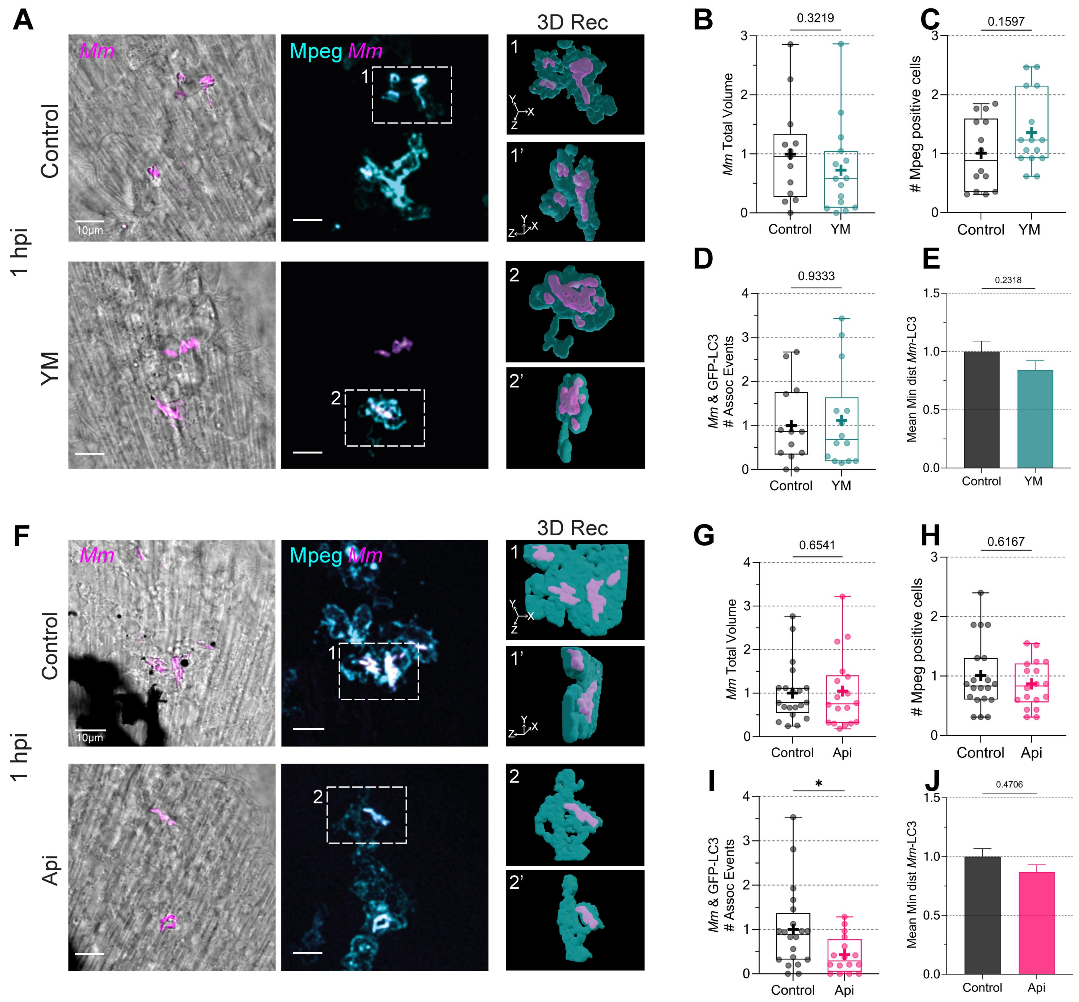

Supplementary Figure 2. PIKfyve inhibition does not affect *Mm* phagocytosis**.** Double transgenic *(CMV:GFP-LC3 / mpeg1.1:mCherryF)* 72 hpf zebrafish larvae, labeling LC3 protein and macrophages (cyan), were incubated in YM201636 (YM) 10 µM or Apilimod (Api) 2 µM. After two hours, the larvae were infected with 100 CFU of E2-Crimson-labelled *Mm* (magenta). Samples were fixed at 1hpi, and CLSM imaging was performed at the ROI in the tail fin. **(A)** and **(F)** Representative fluorescent images and 3D reconstruction of the highlighted areas. Scale bar inset 10 µm. **(B)** and **(G)** *Mm* total volume**.** Data points correspond to the sum of volumes per FOV as a fraction of the control. **(C)** and **(H)** Number of Mpeg positive cells. Data points correspond to the number of cells per FOV as a fraction of the control. **(D)** and **(I)** Number of *Mm*-LC3 association events. Data points correspond to the sum of association events per FOV as a fraction of the control. The results were graphed in box plots from min to max, mean was displayed as '+'. Statistical significance was measured by Mann-Whitney test. **(E)** and **(J)** Mean minimum *Mm*-LC3 distance. Columns correspond to the measured $\mathbf{d_{min}}$ per FOV as a fraction of the control. The results were graphed in a column graph and represented as mean ± SEM. Statistical significance was measured by Mann-Whitney test. YM: N = 3 and n = 5; Api: N = 3 and n = 6. * : $p \leq 0.05$, ** : $p \leq 0.01$, *** : $p \leq 0.001$, **** : $p \leq 0.0001$. $\mathbf{d_{min}}$: minimum distance; FOV: Field of View; Hpf: hours post-fertilization; hpi: hours post-infection. ROI: region of interest.

Classified as Internal | Intern

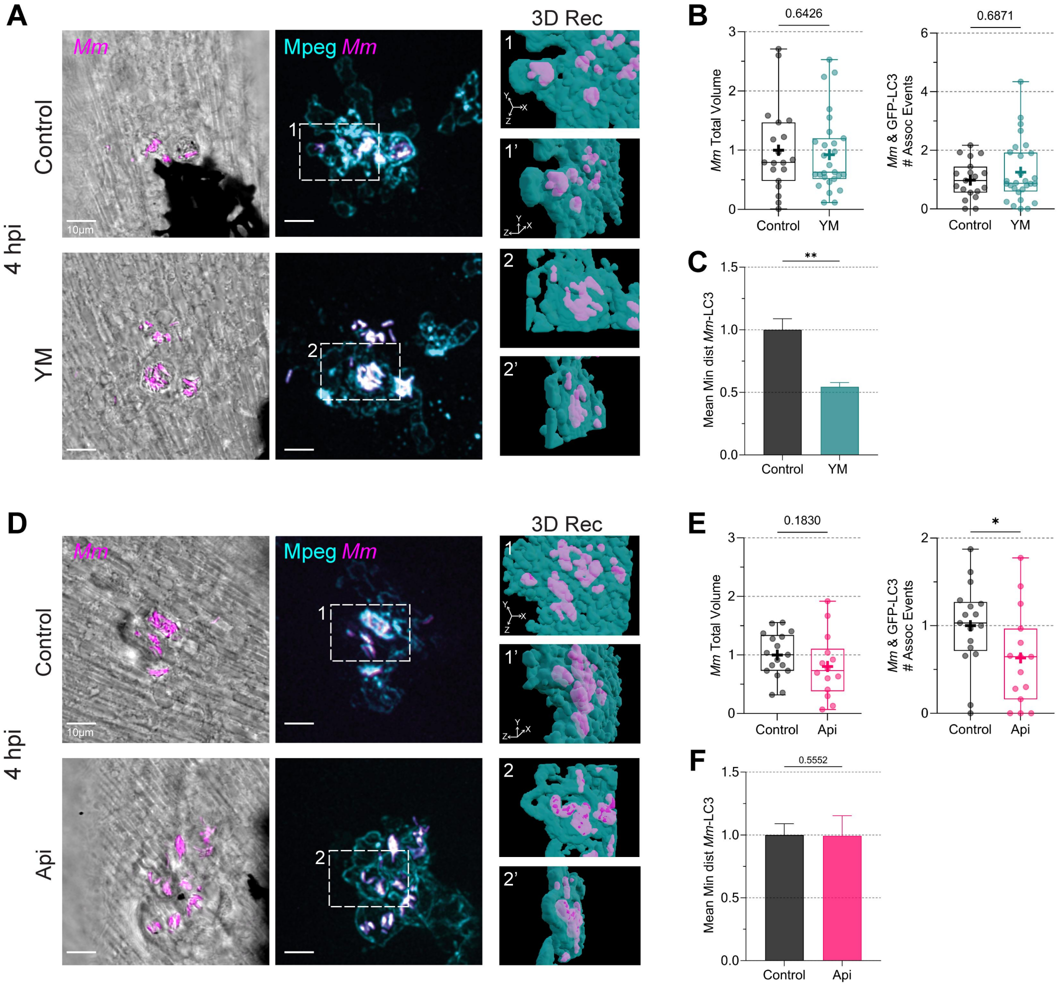

Supplementary Figure 3. Apilimod reduces the number of *Mm*-LC3 association events. Double transgenic *(CMV:GFP-LC3 / mpeg1.1:mCherryF)* 72 hpf zebrafish larvae, labeling LC3 protein and macrophages (cyan), were incubated in YM201636 (YM) 10 µM or Apilimod (Api) 2 µM. After two hours, the larvae were infected with 100 CFU of E2-Crimson-labelled *Mm* (magenta). Samples were fixed at 4 hpi, and CLSM imaging was performed at the ROI in the tail fin. **(A)** and **(D)** Representative fluorescent images and 3D reconstruction of the highlighted areas. Scale bar inset 10 µm. **(B)** and **(E)** *Mm* total volume**.** Data points correspond to the sum of volumes per FOV as a fraction of the control. Number of *Mm*-LC3 association events. Data points correspond to the sum of association events per FOV as a fraction of the control. The results were graphed in box plots from min to max, mean was displayed as '+'. Statistical significance was measured by Mann-Whitney test. **(C)** and **(F)** Mean minimum *Mm*-LC3 distance. Columns correspond to the measured $\mathbf{d_{min}}$ per FOV as a fraction of the control. The results were graphed in a column graph and represented as mean ± SEM. Statistical significance was measured by Mann-Whitney test. YM: N = 3 and n = 5; Api: N = 3 and n = 6. * : $p \leq 0.05$, ** : $p \leq 0.01$, *** : $p \leq 0.001$, **** : $p \leq 0.0001$. $\mathbf{d_{min}}$: minimum distance; FOV: Field of View; Hpf: hours post-fertilization; hpi: hours post-infection. ROI: region of interest.

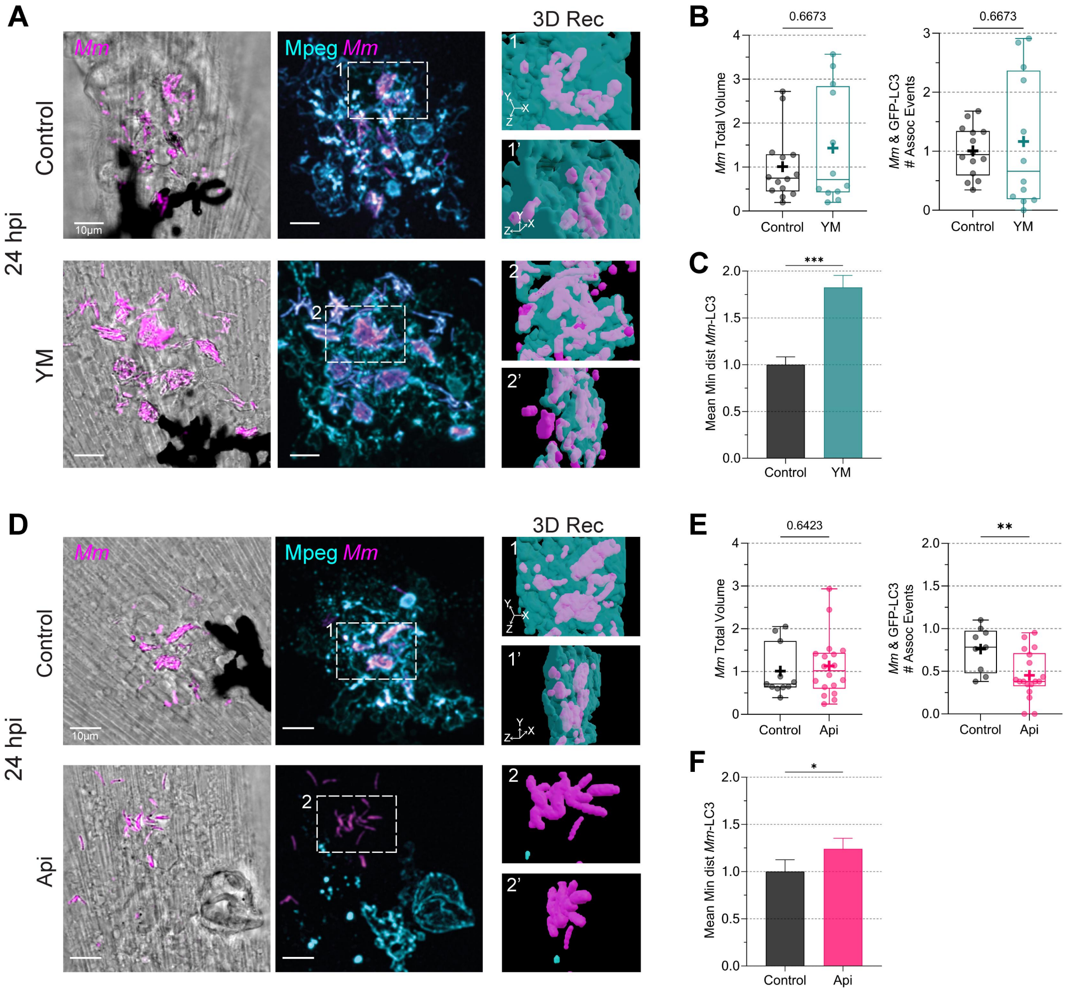

Supplementary Figure 4. PIKfyve inhibition results in the release of *Mm* from macrophages. Double transgenic *(CMV:GFP-LC3 / mpeg1.1:mCherryF)* 72 hpf zebrafish larvae, labeling LC3 protein and macrophages (cyan), were incubated in YM201636 (YM) 10 µM or Apilimod (Api) 2 µM. After two hours, the larvae were infected with 100 CFU of E2-Crimson-labelled *Mm* (magenta). Samples were fixed at 24 hpi, and CLSM imaging was performed at the ROI in the tail fin. **(A)** and **(D)** Representative fluorescent images and 3D reconstruction of the highlighted areas. Scale bar inset 10 µm. **(B)** and **(E)** *Mm* total volume**.** Data points correspond to the sum of volumes per FOV as a fraction of the control. Number of *Mm*-LC3 association events. Data points correspond to the sum of association events per FOV as a fraction of the control. The results were graphed in box plots from min to max, mean was displayed as '+'. Statistical significance was measured by Mann-Whitney test. **(C)** and **(F)** Mean minimum *Mm*-LC3 distance. Data points are measured $\mathbf{d_{min}}$ per FOV as a fraction of the control. The results were graphed in a column graph and represented as mean ± SEM. Statistical significance was measured by Mann-Whitney test. YM: N = 3 and n = 5; Api: N = 3 and n = 6. * : $p \leq 0.05$, ** : $p \leq 0.01$, *** : $p \leq 0.001$, **** : $p \leq 0.0001$. $\mathbf{d_{min}}$: minimum distance; FOV: Field of View; Hpf: hours post-fertilization; hpi: hours post-infection. ROI: region of interest.

Classified as Internal | Intern

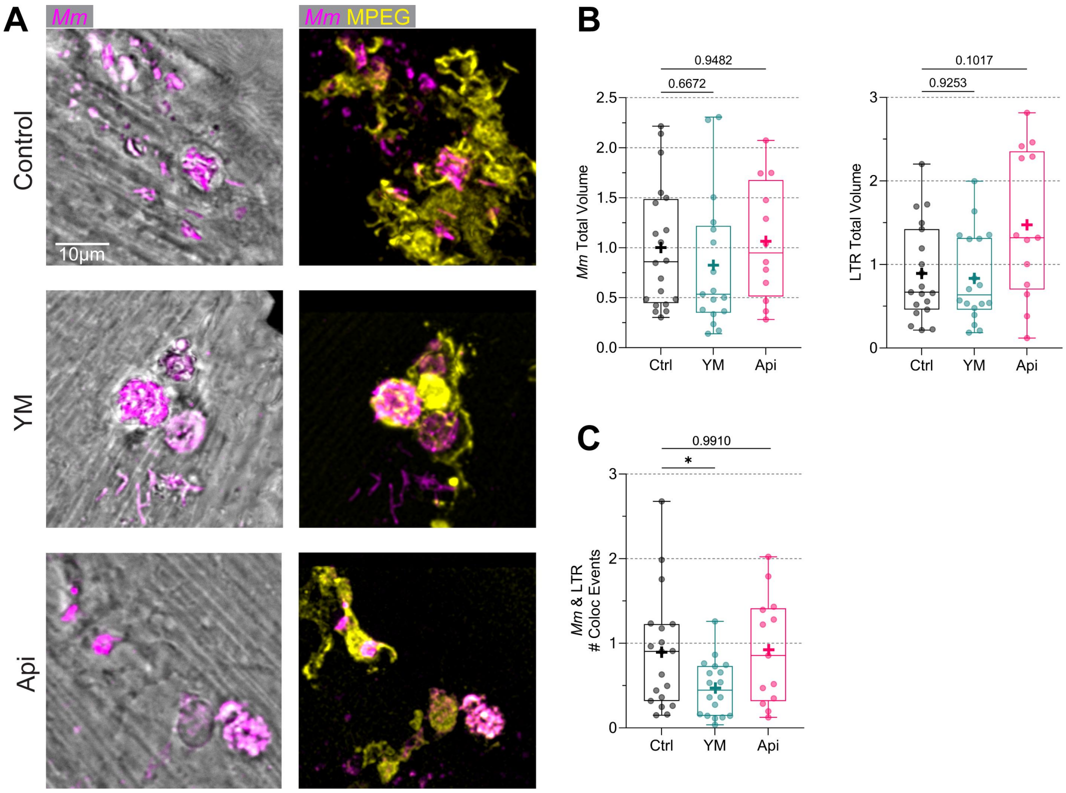

Supplementary Figure 5. PIKfyve inhibition hinders *Mm*-containing vesicles maturation. Transgenic *(mpeg1:EGFP)* 72 hpf zebrafish larvae, labeling macrophages (yellow), were incubated in YM201636 (YM)10 µM or Apilimod (Api) 2 µM. After two hours, the samples were infected with 100 CFU of E2-Crimson-labelled *Mm* (magenta). Samples were incubated for 1 hour in LTR staining (cyan) and fixed at 4hpi. CLSM imaging was performed at the ROI in the tail fin. **(A)** Representative fluorescent images. Scale bar inset 10 µm. **(B)** *Mm* total volume**.** Data points correspond to the sum of volumes per FOV as a fraction of the control. LTR total volume. Data points correspond to the sum of structures and volumes per FOV as a fraction of the control. **(C)** Calculation of *Mm*-LTR number of colocalization events. Data points correspond to the sum of colocalization events per FOV as a fraction of the control. The results were graphed in box plots from min to max, and the mean was displayed as '+'. Statistical significance was measured by Kruskal-Wallis and Dunn's multiple comparisons tests. YM: N = 3 and n = 6; Api: N = 3 and n = 5. * : $p \leq 0.05$, ** : $p \leq 0.01$, *** : $p \leq 0.001$, **** : $p \leq 0.0001$. FOV: Field of View; Hpf: hours post-fertilization; hpi: hours post-infection; LTR: LysoTracker staining; ROI: region of interest.

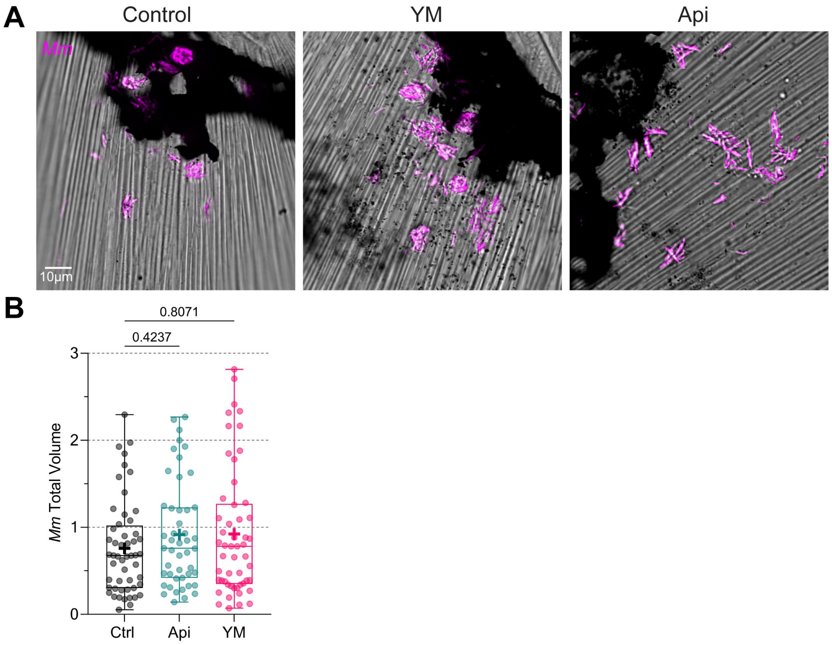

Supplementary Figure 6. PIKfyve inhibition facilitates *Mm*-infected cells death. Transgenic *(mpeg1:EGFP)* 72 hpf zebrafish larvae were incubated in YM201636 10 µM or Apilimod 2 µM. After two hours, samples were infected with 100 CFU of E2-Crimson-labelled *Mm* (magenta). Larvae were fixed at 24hpi, after which the TUNEL assay was performed. Samples were imaged by CLSM at the ROI in the tail fin. **(A)** Representative fluorescent images. Scale bar 10 µm. **(B)** *Mm* total volume**.** Data points correspond to the sum of volumes per FOV as a fraction of the control. The results were graphed in box plots from min to max, and the mean was displayed as '+'. Statistical significance was measured by Kruskal-Wallis and Dunn's multiple comparisons tests. YM: N = 7 and n = 5; Api: N = 6 and n = 5. * : $p \leq 0.05$, ** : $p \leq 0.01$, *** : $p \leq 0.001$, **** : $p \leq 0.0001$. FOV: Field of View; Hpf: hours post-fertilization; hpi: hours post-infection. ROI: region of interest.

Classified as Internal | Intern
